# A dataset for examining trends in publication of new Australian insects

**DOI:** 10.3897/BDJ.2.e1160

**Published:** 2014-07-07

**Authors:** Robert Evan Mesibov

**Affiliations:** † Queen Victoria Museum and Art Gallery, Launceston, Australia

**Keywords:** Taxonomy, Insecta, Australia

## Abstract

Australian Faunal Directory data were used to create a new, publicly available dataset, nai50, which lists 18318 species and subspecies names for Australian insects described in the period 1961–2010, together with associated publishing data. The number of taxonomic publications introducing the new names varied little around a long-term average of 70 per year, with ca 420 new names published per year during the 30-year period 1981–2010. Within this stable pattern there were steady increases in multi-authored and 'Smith in Jones and Smith' names, and a decline in publication of names in entomology journals and books. For taxonomic works published in Australia, a publications peak around 1990 reflected increases in museum, scientific society and government agency publishing, but a subsequent decline is largely explained by a steep drop in the number of papers on insect taxonomy published by Australia's national science agency, CSIRO.

## Introduction

This paper examines trends in publication of new Australian insects over the past 50 years. It is based on 'nai50' (Suppl. material 1), a dataset compiled by the author and freely available at Zenodo (https://zenodo.org/record/10481; http://dx.doi.org/10.5281/zenodo.10481) and as a supplement to this paper. The raw materials for nai50 were names lists for insects from the Australian Faunal Directory (AFD). The AFD is an online resource compiled by taxon specialists, and is maintained and updated by the Department of the Environment, Australia (http://www.environment.gov.au/biodiversity/abrs/online-resources/fauna/afd/home).

The nai50 dataset is based on a snapshot of AFD insect data in early 2014, and will not be updated. It should therefore not be used as a substitute for the AFD as a source of taxonomic information on Australian insects. Another reason not to use nai50 as a taxonomic resource is that the AFD contains errors and formatting inconsistencies (see below). While I tried to correct as many of these as possible in nai50, I may have inadvertently introduced new errors.

## Geographic coverage

### Description

Australia

## Usage rights

### Use license

Creative Commons CCZero

## Data resources

### Data package title

nai50

### Resource link

https://zenodo.org/record/10481

### Alternative identifiers

New Australian insects 1961–2010 from Australian Faunal Directory

### Number of data sets

1

### Data set 1.

#### Data set name

nai50

#### Data format

tab-separated text table

#### Number of columns

22

#### Character set

UTF-8

#### Download URL

https://zenodo.org/record/10481; http://dx.doi.org/10.5281/zenodo.10481

#### Description

6.3 MB plain-text, tab-separated table with 22 columns and 18319 rows (including header row)

**Data set 1.** 

Column label	Column description
AFD_higher_taxon	AFD names list from which I sourced the species or subspecies name
Order	Insect order in which the species or subspecies is placed
Genus	Genus name
Subgenus	Subgenus name (if any)
Species	Species name
Subspecies	Subspecies name (if any)
Name_type	Valid, synonym or replacement name
Rank	Species or subspecies
Orig_combination	Original combination? (Y or N)
Author	Taxonomic author
Year	Year of publication of name
Full_author_name	Taxonomic author(s) for the species or subspecies name, e.g. 'Bellamy, C.L. & Williams, G.A.'
No_auths	Number of taxonomic authors for the species or subspecies name
A_in_B	yes/no, for separate taxonomic name authorship within a publication, e.g. 'Peterson, M. in Peterson, M. & Bellamy, C.L.'
Pub_ID	a unique serial number for the publication in which the species or subspecies was described
Citation	Full reference citation
Pub_type	Article in Journal, Book, Chapter in Book or Section in Article
Pub_country	The country in which the work describing the species or subspecies was published
Pub_ento	yes/no, for whether the work describing the species or subspecies was published in an entomological journal or book
J_title	Journal title from Citation field, or 'na' for Book and Chapter in Book
A_publr_class	For works published in Australia, whether the publisher was a government agency, a museum, a scientific society or 'other', e.g. a private individual; 'na' for works not published in Australia
A_publr	For works published in Australia, the name of the publisher, e.g. CSIRO; 'na' for works not published in Australia

## Additional information

### AFD limitations

At the time of this study, AFD metadata noted that taxonomic information on Chrysomeloidea, Heteroptera, Orthorrhapha, Staphylinoidea and Tenebrionoidea was incomplete. Further, some of the 'complete' insect groups in the AFD were not up to date, although the currency of the lists was not always clear from the metadata. For example, the Aleyrodoidea metadata gives a compilation date of 31 December 2001, yet the names list for this taxon includes species from a 2012 publication.

Another limitation concerns introduced species, which I wanted to exclude from my compilation of new Australian insects. The AFD database can export a list of introduced insects through its 'Advanced Search' function (http://www.environment.gov.au/biodiversity/abrs/online-resources/fauna/afd/search/advanced), but membership of the list depends on compilers annotating a name as 'introduced', and I was advised by AFD staff that not all compilers have done so (C. Geromboux, in litt. 13 April 2014).

### Date range

I chose the date range 1961–2010 to represent 'the last 50 years'. The slight backdating increased the chances that AFD names lists were up to date for the purposes of this study, and also allowed me to class the data by decades, i.e. 1960s, 1970s etc. (I follow the convention that decades begin with a year ending in '1', since there was no year '0' in the Western calendar.)

### AFD downloads and initial processing

In early 2014, the AFD allowed for export of names lists for any higher taxa, e.g an insect order or superfamily, as comma-separated value (CSV) files. A download limit prevented export of a names list for all insects, so I chose the largest higher taxa for which export was possible. I downloaded most of the lists in March 2014, more in April 2014 while AFD staff addressed an access problem on the AFD Web portal, and the remainder in June 2014.

The CSV files were merged, converted to a tab-separated value (TSV) file and processed as described below. Processing was done on the command line using GNU/Linux utilities and the AWK programming language, or in Gnumeric spreadsheet software. The merged AFD names lists were filtered to:

- select names with the rank of species and subspecies and with publication years in the range 1961–2010, inclusive,
- delete names which AFD compilers had annotated as misspellings, unnecessary emendations, nomina nuda, status unresolved and literature records,
- delete duplicate names,
- delete replacement names for species and subspecies described before 1961, and
- delete names of species and subspecies introduced to Australia, using as reference a list of 472 names generated in April 2014 with the AFD 'Advanced Search' function (see above, 'AFD limitations')

Of the original fields in the AFD names lists, I retained GENUS, SUBGENUS, SPECIES, SUBSPECIES, NAME_TYPE, RANK, AUTHOR, YEAR, ORIG[INAL]_COMBINATION, PUB_PUB_TYPE and PUB_PUB_FORMATTED. Field contents were modified as follows:

- Author, year, original combination and publication type were corrected as required (see below, Data cleaning).
- Name_type was modified to hold 'Valid', 'Replaced by [name and author and year]' or 'Synonym of [name and author and year]'. 'Replaced by... ' entries in nai50 contain replacement names for species or subspecies described in the period 1961–2010; valid names with minor emendations (e.g. a published change of species epithet to correct lack of agreement in gender with genus) were left as emended. 'Synonym of... .' entries contain valid names for species and subspecies described in the period 1961–2010 and subsequently placed in synonymy. In both cases (replacement and synonymy) the information in nai50's Name_type field comes from the relevant AFD names list.
- Rank was modified to reflect the rank of the taxon as described; e.g. if an author described a subspecies and the name was later synonymised with a species, the Rank field in nai50 contains 'Subspecies', not 'Species'.
- Citation (PUB_PUB_FORMATTED in AFD) was modified to remove AFD markup, correct errors and make formatting more consistent for unique publications (i.e., I made the text string for a particular publication the same for all entries where it appeared).

### Further processing

In the following list of fields in nai50, the fields I added are marked with an asterisk and described in bracketed notes:

*AFD_higher_taxon (AFD names list from which I sourced the species or subspecies name)

*Order (insect order in which the species or subspecies is placed)

Genus

Subgenus

Species

Subspecies

Name_type

Rank

Orig_combination [Y or N]

Author

Year

*Full_author_name (taxonomic author(s) for the species or subspecies name, e.g. 'Bellamy, C.L. & Williams, G.A.')

*No_auths (number of taxonomic authors for the species or subspecies name)

*A_in_B (yes/no, for separate taxonomic name authorship within a publication, e.g. 'Peterson, M. in Peterson, M. & Bellamy, C.L.')

*Pub_ID (a unique serial number for the publication in which the species or subspecies was described)

Citation

Pub_type (Article in Journal, Book, Chapter in Book or Section in Article)

*Pub_country (the country in which the work describing the species or subspecies was published)

*Pub_ento (yes/no, for whether the work describing the species or subspecies was published in an entomological journal or book)

*J_title (journal title from Citation field, or 'na' for Book and Chapter in Book)

*A_publr_class (for works published in Australia, whether the publisher was a government agency, a museum, a scientific society or 'other', e.g. a private individual; 'na' for works not published in Australia)

*A_publr (for works published in Australia, the name of the publisher, e.g. CSIRO; 'na' for works not published in Australia)

### Data cleaning

I used programmatic checks on nai50 to find AFD omissions, errors and formatting inconsistencies, which were numerous. Omissions and errors were corrected with the aid of original publications and online resources compiled by taxon specialists. I am not a taxon specialist for any insect group, so the taxonomic errors I corrected were mainly those detected by programmatic checks, such as comparing the AFD fields YEAR and PUB_PUB_YEAR, and AUTHOR and PUB_PUB_AUTHOR for all names. For checking the link between name and citation, the BioNames project (http://bionames.org/) was particularly helpful. Bibliographic data on publications came from Worldcat (https://www.worldcat.org/) and the National Library of Australia (http://trove.nla.gov.au/).

### Analysis

Data summaries and working tables were generated from nai50 using AWK commands. For charting and consistency checks, working tables were imported into Gnumeric spreadsheet software. The statistics in the analysis are purely descriptive.

### Overview and summary statistics

The dataset 'nai50' is a 6.3 MB plain-text, tab-separated table with 22 columns and 18319 rows (including header row). It contains the names of 17905 species (97.7% of all names) and 413 subspecies (2.3%) of Australian insects described in the period 1961–2010. The 18318 total may include some introduced insects and is likely to omit some recently described species and subspecies, as well as species and subspecies in data gaps in the AFD (see 'AFD limitations' above). However, I regard the total as large enough for the primary purpose of this study, which is to identify trends in publication.

The 18318 species and subspecies in nai50 were described in 3628 publications by ca 1460 taxonomic authors. The latter number is tentative because it is not always clear from a citation alone whether 'Smith, A.' is the same author as 'Smith, A.B.'. For the same reason, I did not identify individual taxonomists in the list of taxonomic authors. Note that the 1460 figure counts 'Smith, A. & Jones, B.' and 'Jones, B. & Smith, A.' as different taxonomic authors, but 'Smith, A.' and 'Smith, A. in Jones, B. & Smith, A.' as the same taxonomic author.

**Figure 1.**
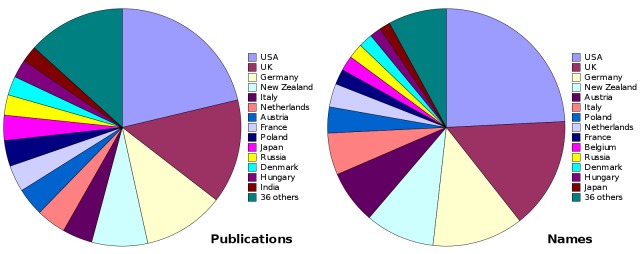
New Australian insects in nai50 (1961–2010) by country of publication other than Australia, tallied by number of publications (left) and number of species and subspecies (right) (Suppl. material 2).

The 3628 taxonomic works were published in 51 countries, with Australia accounting for 1499 of the publications (41.3%) and 9796 of the species and subspecies names (53.5%). Publications in the USA, UK, Germany and New Zealand contributed more than half of the remaining taxonomic works and names (Fig. 1).

Most new names were published in journal articles (16397; 89.5%), followed by books (1652; 9.0%), chapters in books (256; 1.4%) and sections of articles (13; <0.1%).

The AFD recorded 523 of the 17905 species names (2.9%) and 68 of the 413 subspecies names (16.5%) as synonymised. Synonymised names have been included in the trend analysis below.

**Figure 2.**
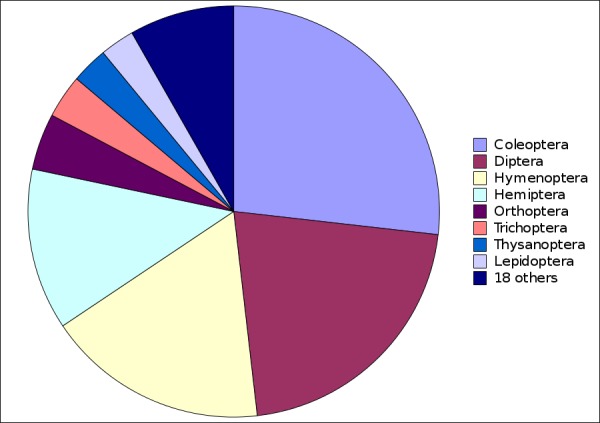
New Australian insect names in nai50 (1961–2010) by order (Suppl. material 3).

The 18318 species and subspecies were in 26 orders, with Coleoptera, Diptera, Hymenoptera and Hemiptera contributing more than three-quarters of all names (Fig. 2). Publications were almost entirely 'order-loyal'; only four of the 3628 works (naming 29 species or subspecies) included new names from more than one insect order.

### Trends in authorship

A single publication can contain names with different numbers of taxonomic authors, e.g. 'Smith in Jones & Smith, 1998' for one name and 'Jones & Smith, 1998' for another. The following trends count publications in a year, but nine of the 3628 publications have been double-counted for the reason just mentioned.

**Figure 3.**
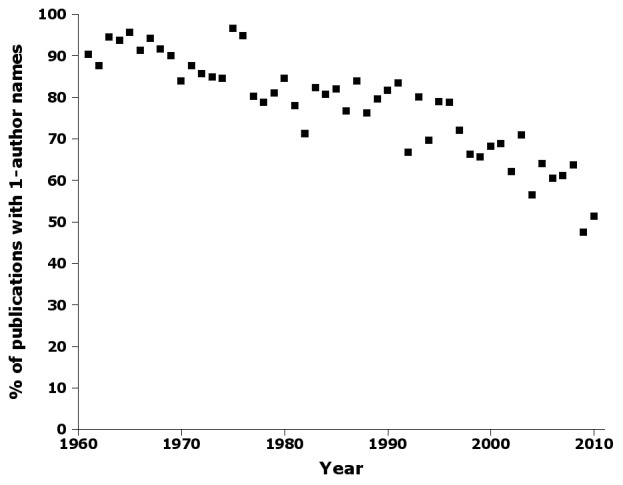
Percentage of publications by year in nai50 with single-author names, e.g. 'Smith, 1998' (Suppl. material 4).

There was a marked and steady decline in the proportion of publications with single-authored names (Fig. 3), from ca 90% of publications in the 1960s to about half of all publications today.

**Figure 4.**
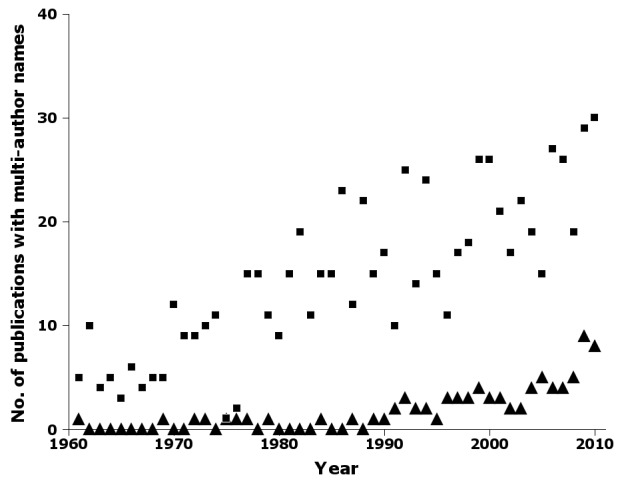
Number of publications by year in nai50 with two-author (squares) and three-author (triangles) names, e.g. 'Smith & Jones, 1998' and 'Smith, Jones & Brown, 1998' (Suppl. material 5).

The number of publications introducing names with two taxonomic authors increased steadily over the 50-year sampling period, with an increase in three-author names starting in the 1990s (Fig. 4). A six-author name was published in 1998, and a seven-author name in 2005.

Having more taxonomic authors, however, did not lead to correspondingly greater taxonomic productivity, i.e. more new names per publication, and single taxonomic authors consistently introduced the majority of new Australian species and subspecies (Table 1).

**Figure 5.**
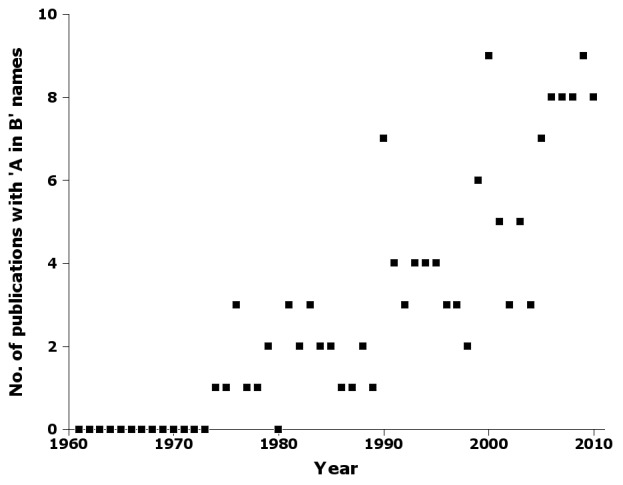
Number of publications by year in nai50 with 'A in B' names, e.g. 'Smith in Jones & Smith, 1998' (Suppl. material 6).

Another strong trend in authorship was a steady increase in the number of publications containing names with 'A in B' authorship, e.g. 'Smith in Jones & Smith, 1998' (Fig. 5). The first 'A in B' authorship in nai50 was in 1974.

### Trends in publishing

**Figure 6.**
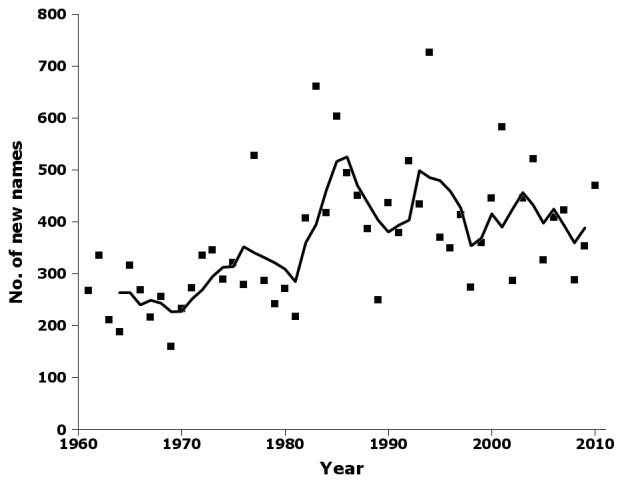
Number of new species and subspecies names by year in nai50, showing raw data (squares) and 5-year moving average (solid line) (Suppl. material 7).

Following a strong increase at the beginning of the 1980s, the number of new species and subspecies varied around an average of ca 420 per year for the last 30 years of the sampling period, with an average of 430 in the 1980s, 427 in the 1990s and 410 in the 2000s (Fig. 6).

**Figure 7.**
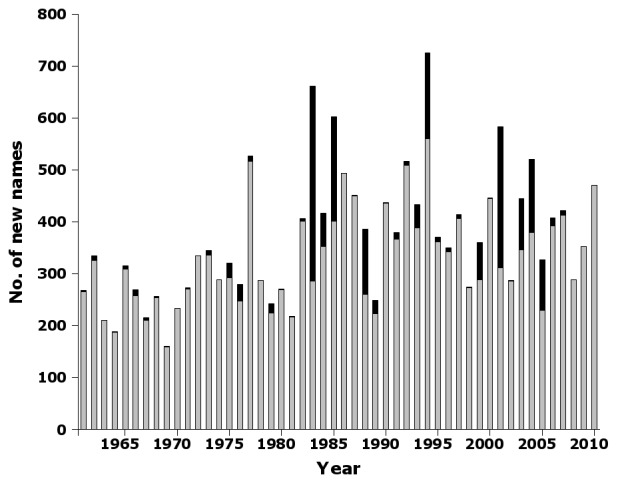
Number of new species and subspecies names by year in nai50 published in books (black) and journal articles (grey) (Suppl. material 8).

**Figure 8.**
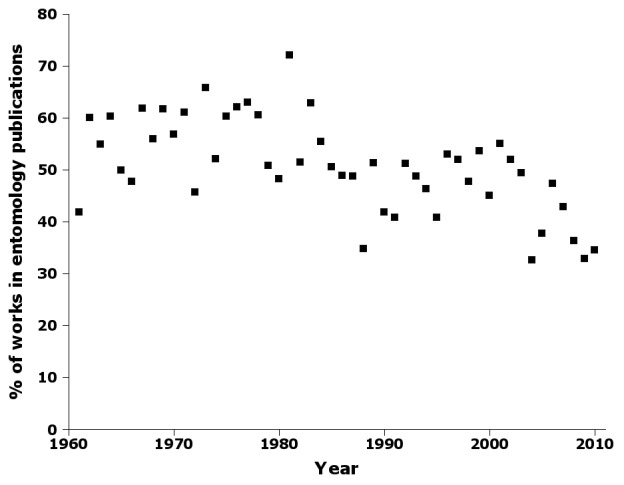
Percentage of publications by year in nai50 in entomology books and journals (Suppl. material 9).

The publication of new names in journals was fairly steady over the 50-year sampling period (Fig. 7) New names also appeared fairly steadily in books, but books only first became significant outlets for new names in the 1980s (Fig. 7). Beginning in the mid-1980s, the proportion of publications with new Australian insect names which were devoted to entomology abruptly declined, reaching a 50-year low of about one-third of publications in 2009 (Fig. 8).

There was a small increase in the average number of new names per publication (Table 2), but little change over the last 30 years. Much of the increase was in non-Australian publications; the average number of new names per publication in Australia varied little in 50 years (Table 2). Average new names per publication was greater in non-Australian publications than in Australian publications in nine of the 50 years, and five of the nine were in the 2000s.

**Figure 9.**
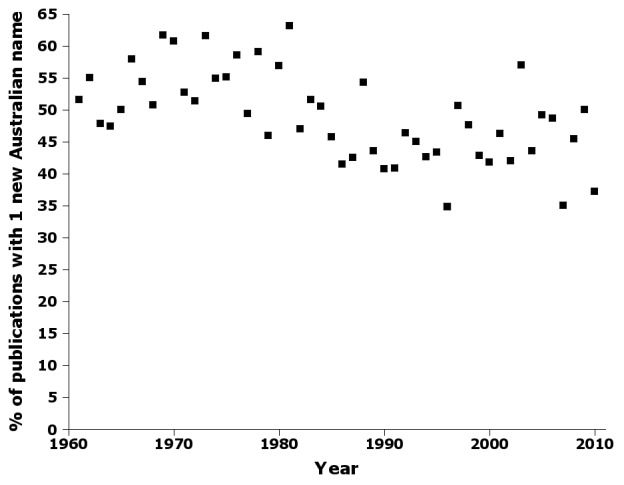
Percentage of publications by year in nai50 containing only one new species or subspecies name (Suppl. material 10).

The proportion of publications containing only one new Australian insect name declined, but not dramatically, from ca 55% in the 1960s to ca 45% in the 2000s (Fig. 9). Note that this is not the same as the proportion of publications containing a single new name; a publication reviewing a regional fauna, for example, might contain new names for many species or subspecies, only one of which is Australian.

### Trends in Australian publishing

**Figure 10.**
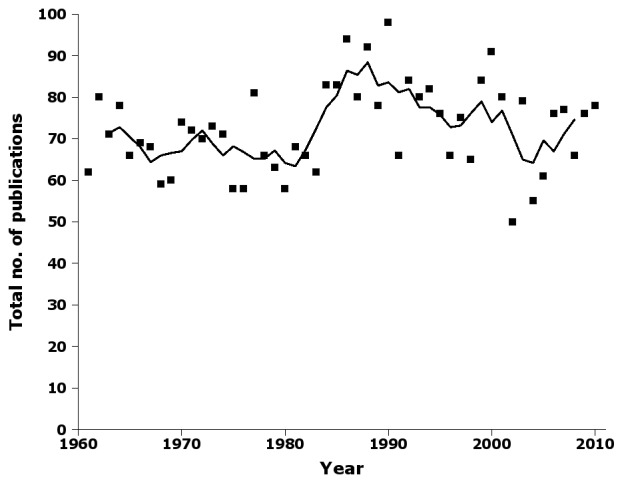
Number of publications by year in nai50, showing raw data (squares) and 5-year moving average (solid line) (Suppl. material 11).

**Figure 11.**
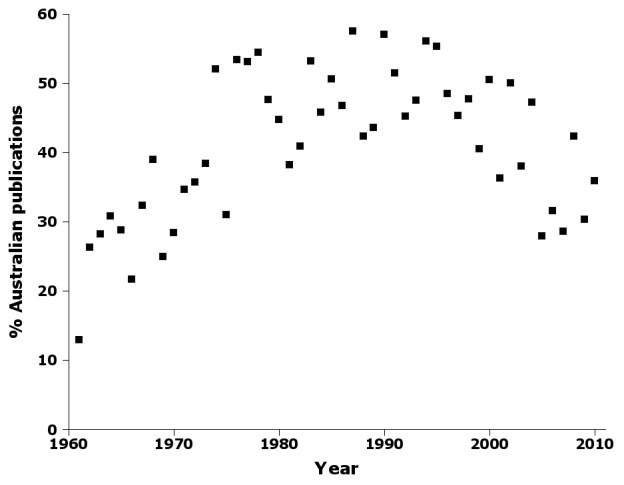
Percentage of Australian publications by year in nai50 (Suppl. material 12).

The number of publications containing new Australian insect names varied surprisingly little around a long-term average of ca 70 publications per year (Fig. 10).  Higher values around 1990 and lower values more recently are largely explained by a distinct peak in Australian publications (Fig. 11). At the peak, a little more than half the taxonomic works containing new names were Australian publications, while the proportion had dropped to about a third by the end of the 2000s. To explore this trend more closely, I categorised Australian publications by publisher and publisher class (see 'Further processing', above). Overall statistics are given in Table 3.

**Figure 12.**
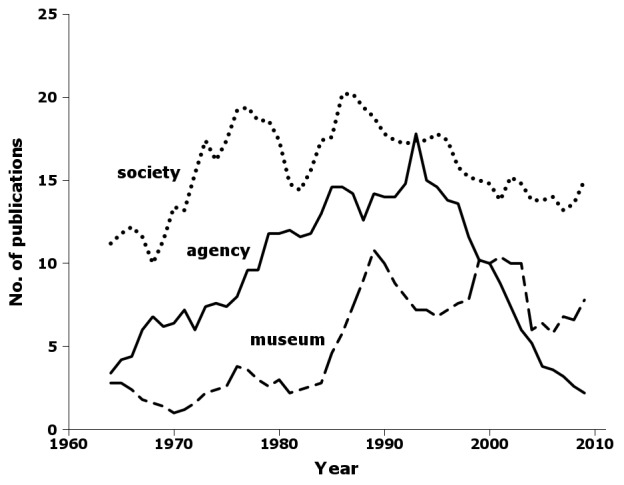
Five-year moving average of Australian publications in nai50 by year and publisher class: agency (solid line), museum (dashed line) and society (dotted line) (Suppl. material 13).

Five-year moving averages for the three main Australian publisher classes (Fig. 12) show that the 1990 peak in Australian publications reflected broadly synchronous peaks in agency, museum and society publishing. While museum and society publishing later declined only slightly from their peaks, agency publishing dropped precipitously (Fig. 12). All but two of the 438 agency publications from 1961–2010 were produced by CSIRO, the Australian government's national science agency, and the 436 CSIRO publications contained 5366 new species and subspecies, or 29% of the total for the 50-year period.

**Figure 13.**
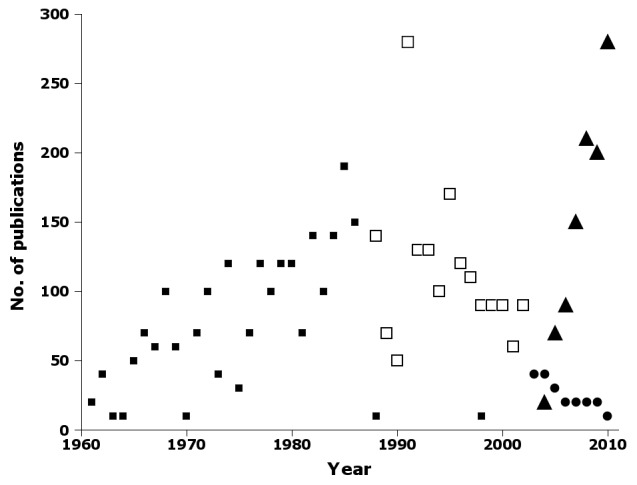
Number of publications by year in nai50 in *Australian Journal of Zoology* (filled squares), *Invertebrate Taxonomy* (open squares), *Invertebrate Systematics* (circles) and *Zootaxa* (triangles) (Suppl. material 14).

**Figure 14.**
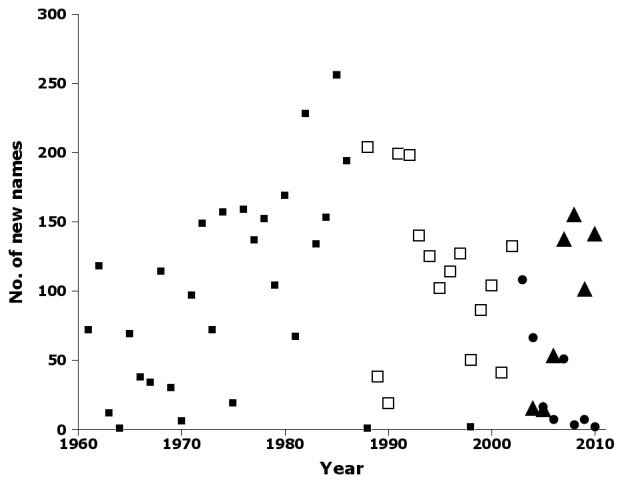
Number of new species and subspecies names by year in nai50 in *Australian Journal of Zoology* (filled squares), *Invertebrate Taxonomy* (open squares), *Invertebrate Systematics* (circles) and *Zootaxa* (triangles) (Suppl. material 14).

Tracking publications (Fig. 13) and names (Fig. 14) in three of the CSIRO journals reveals an interesting pattern. *Australian Journal of Zoology* published an increasing number of papers and names until 1986. In 1987, CSIRO published the first issue of *Invertebrate Taxonomy*, and only two papers and three names were subsequently published in *Australian Journal of Zoology*. Papers and names declined in *Invertebrate Taxonomy* until 2002, when CSIRO renamed the journal *Invertebrate Systematics* and discouraged purely taxonomic submissions. Papers and names in *Invertebrate Systematics* dropped steeply during the 2000s, a period which saw steady growth in papers and names in the New Zealand journal *Zootaxa* (Figs 13, 14).

### Discussion

The nai50 dataset provides an objective basis for identifying long-term research and publishing trends in Australian entomology, and is readily extendable. Since the dataset is now in the public domain, interested users are welcome to keep it up to date, extend it backwards in time and add new fields, such as author age and affiliation at time of publication.

Users are also welcome to search for and correct errors, which are undoubtedly still present in nai50. For every hour exploring and analysing nai50, I spent several hours detecting, investigating and correcting omissions, errors and formatting inconsistencies in AFD data. AFD data validation, both at the time of data entry by specialist compilers and later by AFD staff, could usefully be extended and improved. In correspondence with the author, AFD staff have said they are aware of the data cleaning issues in AFD and hope to address them more effectively when additional resources are made available to the project.

## Supplementary Material

Supplementary material 1 nai50 Data type: bibliographic data
Brief description: 6.3 MB plain-text, tab-separated table with 22 columns and 18319 rows (including header row).
File: oo_8235.csv Mesibov

Supplementary material 2 Figure 1 data Data type: bibliographic
Brief description: Numbers of publications and new names from the nai50 dataset, by country.
File: oo_8237.tsv Robert Mesibov

Supplementary material 3 Figure 2 data Data type: bibliographic
Brief description: Numbers of new insect names in nai50 dataset, by order.
File: oo_8239.tsv Robert Mesibov

Supplementary material 4 Figure 3 data Data type: bibliographic
Brief description: Percentage of publications by year in nai50 with single-author names.
File: oo_8240.tsv Robert Mesibov

Supplementary material 5 Figure 4 data Data type: bibliographic
Brief description: Number of publications by year in nai50 with two-author (squares) and three-author (triangles) names.
File: oo_8241.tsv Robert Mesibov

Supplementary material 6 Figure 5 data Data type: bibliographic
Brief description: Number of publications by year in nai50 with 'A in B' names.
File: oo_8242.tsv Robert Mesibov

Supplementary material 7 Figure 6 data Data type: bibliographic
Brief description: Number of new species and subspecies names by year in nai50, with 5-year moving average.
File: oo_8243.tsv Robert Mesibov

Supplementary material 8 Figure 7 data Data type: bibliographic
Brief description: Number of new species and subspecies names by year in nai50 published in books and journal articles.
File: oo_8244.tsv Robert Mesibov

Supplementary material 9 Figure 8 data Data type: bibliographic
Brief description: Percentage of publications by year in nai50 in entomology books and journals.
File: oo_8245.tsv Robert Mesibov

Supplementary material 10 Figure 9 data Data type: bibliographic
Brief description: Percentage of publications by year in nai50 containing only one new species or subspecies name.
File: oo_8246.tsv Robert Mesibov

Supplementary material 11 Figure 10 data Data type: bibliographic
Brief description: Number of publications by year in nai50, with 5-year moving average.
File: oo_8247.tsv Robert Mesibov

Supplementary material 12 Figure 11 data Data type: bibliographic
Brief description: Percentage of Australian publications by year in nai50.
File: oo_8248.tsv Robert Mesibov

Supplementary material 13 Figure 12 data Data type: bibliographic
Brief description: Five-year moving average of Australian publications in nai50 by year and publisher class.
File: oo_8249.tsv Robert Mesibov

Supplementary material 14 Figures 13, 14 data Data type: bibliographic
Brief description: Number of publications and new names by year in nai50 in Australian Journal of Zoology, Invertebrate Taxonomy, Invertebrate Systematics and Zootaxa.
File: oo_8250.tsv Robert Mesibov

## Figures and Tables

**Table 1.** Mean new names/publication by decade in nai50 with 1, 2 and 3 taxonomic authors (number of new names).

Decade	1 author	2 authors	3 authors
1960s	3.7 (2323)	2.1 (126)	1.5 (3)
1970s	5.1 (2898)	2.9 (263)	1.3 (8)
1980s	5.5 (3473)	5.1 (844)	1.2 (5)
1990s	5.9 (3316)	4.7 (875)	2.9 (76)
2000s	6.4 (2693)	4.9 (1099)	6.5 (300)
All years	5.2 (14703)	4.4 (3207)	4.7 (392)

**Table 2.** Mean new names/publication by decade in nai50 for Australian, non-Australian and all publications (number of publications).

Decade	Australian	non-Australian	All
1960s	5.8 (188)	2.7 (499)	3.6 (687)
1970s	6.9 (299)	3.0 (371)	4.7 (670)
1980s	6.2 (385)	4.6 (419)	5.4 (804)
1990s	6.9 (375)	4.3 (394)	5.6 (769)
2000s	6.6 (252)	5.5 (446)	5.9 (698)
All years	6.5 (1499)	4.0 (2129)	5.0 (3628)

**Table 3.** Number of publications and number of new names in Australia by publisher class and publisher in nai50, 1961–2010. Publisher abbreviations: AAD = Australian Antarctic Division, ABRS = Australian Biological Resources Study, CSIRO = Commonwealth Scientific and Industrial Research Organisation, QldGovt = Queensland state government, TasGovt = Tasmanian state government, AM= Australian Museum, MAGNT = Museums and Art Galleries of the Northern Territory, NMV = Museum Victoria, QM = Queensland Museum, QVMAG = Queen Victoria Museum and Art Gallery, SAM = South Australian Museum, WAM = Western Australian Museum, AustEntSoc = Australian Entomological Society, BOIC = Butterflies and Other Invertebrates Club, EntSocNSW = Entomological Society of New South Wales, EntSocQld = Entomological Society of Queensland, FieldNatsSA = Field Naturalists Society of South Australia, FieldNatsVic = Field Naturalists Club of Victoria, FieldNatsWA = Western Australian Naturalists Club, RoySocQld = Royal Society of Queensland, RoySocSA = Royal Society of Soyj Australia, RoySocTas = Royal Society of Tasmania, RoySocVic = Royal Society of Victoria, RoySocWA = Royal Society of Western Australia, RoyZooSocNSW = Royal Zoological Society of New South Wales, UniQld = University of Queensland.

Publisher class	Publisher	No. of publications	No. of new names	Publication
agency	AAD	1	6	Australian New Zealand Antarctic Research Expedition Reports
agency	ABRS	1	9	book
agency	CSIRO	5	18	Australian Journal of Marine and Freshwater Research
agency	CSIRO	127	1019	Australian Journal of Zoology
agency	CSIRO	87	1725	Australian Journal of Zoology Supplementary Series
agency	CSIRO	21	265	Invertebrate Systematics
agency	CSIRO	172	1679	Invertebrate Taxonomy
agency	CSIRO	24	660	books
agency	QldGovt	5	18	Queensland Journal of Agricultural and Animal Sciences
agency	TasGovt	1	1	book
museum	AM	2	162	Memoirs of the Australian Museum
museum	AM	42	273	Records of the Australian Museum
museum	AM	1	208	Records of the Australian Museum, Supplement
museum	MAGNT	1	8	Occasional Papers of the Northern Territory Museum of Arts and Sciences
museum	MAGNT	14	46	The Beagle, Records of the Museums and Art Galleries of the Northern Territory
museum	NMV	27	284	Memoirs of Museum Victoria
museum	NMV	19	144	Memoirs of the National Museum of Victoria
museum	QM	70	444	Memoirs of the Queensland Museum
museum	QVMAG	2	2	Records of the Queen Victoria Museum
museum	SAM	51	252	Records of the South Australian Museum
museum	SAM	2	119	Records of the South Australian Museum, Monograph Series
museum	WAM	36	122	Records of the Western Australian Museum
museum	WAM	2	8	Records of the Western Australian Museum, Supplement
museum	WAM	1	84	Special Publications of the Western Australian Museum
society	AustEntSoc	122	285	Australian Journal of Entomology
society	AustEntSoc	349	791	Journal of the Australian Entomological Society
society	BOIC	1	1	Butterfly & Other Invertebrates Club Inc. Newsletter
society	EntSocNSW	18	31	General and Applied Entomology
society	EntSocNSW	2	17	Journal of the Entomological Society of Australia
society	EntSocQld	47	69	Australian Entomological Magazine
society	EntSocQld	51	68	Australian Entomologist
society	EntSocQld	27	57	Journal of the Entomological Society of Queensland
society	FieldNatsSA	2	2	South Australian Naturalist
society	FieldNatsVic	5	9	Victorian Naturalist
society	FieldNatsWA	1	1	Western Australian Naturalist
society	LinnSocNSW	60	187	Proceedings of the Linnean Society of New South Wales
society	RoySocQld	8	35	Proceedings of the Royal Society of Queensland
society	RoySocSA	60	493	Transactions of the Royal Society of South Australia
society	RoySocTas	10	33	Papers and Proceedings of the Royal Society of Tasmania
society	RoySocVic	5	13	Proceedings of the Royal Society of Victoria
society	RoySocWA	3	10	Journal of the Royal Society of Western Australia
society	RoyZooSocNSW	3	78	The Australian Zoologist
other	private	2	2	Calodema
other	private	5	51	books
other	UniQld	3	6	University of Queensland Papers, Department of Entomology
other	UniQld	1	1	University of Queensland Papers, Department of Zoology

